# 10-year ASCVD risk is positively correlated with depressive symptoms in a large general population

**DOI:** 10.1186/s12888-019-2114-7

**Published:** 2019-04-26

**Authors:** Guo-Zhe Sun, Ning Ye, Shao-Jun Wu, Ying Zhou, Ying-Xian Sun

**Affiliations:** Department of Cardiovascular Medicine, The First Hospital of China Medical University, 155 Nanjing Street, Heping, Shenyang, 110001 Liaoning China

**Keywords:** 10-year ASCVD risk, Patient health Questionnaire-9, Depressive symptoms

## Abstract

**Background:**

To explore the potential correlation between 10-year atherosclerotic cardiovascular disease (ASCVD) risk and depressive symptoms in a general population.

**Methods:**

A cross-sectional study involving 11,956 permanent residents of Liaoning Province in China ≥35 years of age was conducted. Depressive symptoms were assessed with the Patient Health Questionnaire-9 (PHQ-9) while 10-year ASCVD risk was calculated using the tool suitable for China.

**Results:**

Males had significantly higher 10-year ASCVD risk than females (14.2 ± 10.7% vs. 9.3 ± 9.1%; *P* <  0.001) but lower PHQ-9 score (2.34 ± 3.13 vs. 3.63 ± 4.02; *P* <  0.001). The mean PHQ-9 score increased significantly with advancing 10-year ASCVD risk category in both males (from 2.03 to 2.61; *P* for trend < 0.001) and females (from 3.04 to 4.61; *P* for trend < 0.001), and the increasing trend was more apparent in females (*P* <  0.001). Pearson correlation analyses showed that 10-year ASCVD risk positively correlated with PHQ-9 score in both sexes (*P*s <  0.001). In multivariate linear regression analyses adjusting for confounding risk factors, the independent associations of 10-year ASCVD risk with PHQ-9 score were all significant in the total (β = 2.61; *P* <  0.001), male (β = 1.64; *P* = 0.001), and female subjects (β = 3.71; *P* <  0.001). Further, the interaction analysis proved the impacts of 10-year ASCVD risk on PHQ-9 score were more apparent in females than males (*P*s < 0.001).

**Conclusions:**

The 10-year ASCVD risk was positively associated with depressive symptoms in both males and females, which was more apparent in the latter. These findings provided some novel data about the value of 10-year ASCVD risk in estimating depressive symptoms.

## Background

Nowadays, cardiovascular disease (CVD) has become the leading cause of death and disease burden in China and world-wide [1, 2]. Great efforts have focused on the prevention and treatment of CVD. The Framingham Risk Score has long been proved a strong predictor of developing coronary heart disease (CHD) and cardiovascular events [3, 4]. And it’s used as a simple tool to evaluate the 10-year risk of CHD to inform the initiating of primary prevention. In China, Gu et al. developed and validated the Chinese atherosclerotic cardiovascular disease (ASCVD) risk equation based on the China-PAR project (Prediction for ASCVD Risk in China) in multiple contemporary Chinese cohorts [5]. This equation was suitable for China and popular-used for the prediction of ASCVD risk.

Depression has become a worldwide public health problem, especially in women [6, 7], which contributes to an increased risk of disability [8] and mortality [9]. The prevalence of depression is significantly higher in patients with CVD and its presence increases the risk of adverse cardiovascular events [10]. Furthermore, depression has been proved recently to be an independent risk factor for the incidence of CHD [11] or even ischemic heart disease [12]. Therefore, it’s quite an important issue to define those with high possibility of depression so that we could make some strategies to control depressive symptoms and prevent its increased risk of ASCVD.

However, whether the 10-year ASCVD risk is also associated with depressive symptoms or not has never been reported, even though the prevalence of depression was proved to be apparently higher in patients with CVD [13]. Therefore, the current study was designed to explore the potential correlation between 10-year ASCVD risk and depressive symptoms in a large general Chinese population.

## Methods

A multi-stage, random, stratified, cluster-sampling scheme was performed in this study. The details about research design, data collection and measurements have been described previously [14, 15]. This study was approved by the Ethics Committee of China Medical University, and written consent was obtained from each participant or the proper proxy.

### Study population

A total of 14,016 eligible permanent residents ≥35 years of age were invited to participate in the study, and 11,956 agreed and completed the study with a response rate of 85.3%. The exclusion criteria included pregnancy, malignant tumor and severe mental disorders (for example psychosis).

### The patient health Questionnaire-9 score

In this study, we adopted the Patient Health Questionnaire-9 (PHQ-9) to evaluate depressive disorder, which was widely used in primary health settings as a screening instrument with good reliability and validity [16–18]. Based on the PHQ-9 tool, the total score would range from 0 to 27, and the severity of depressive disorder was then estimated by the level of PHQ-9 score [19]. And in this study, we conducted the analyses using PHQ-9 score as continuous scale.

### 10-year ASCVD risk

The 10-year predicted risk of ASCVD was calculated using the equations suitable for China developed by Gu et al. [5]. In the equations, besides the major risk factors including age, treated or untreated systolic blood pressure (SBP), total cholesterol (TC), high density lipid cholesterol (HDL-C), current smoking, and diabetes mellitus, 4 additional variables including waist circumference, geographic region, urbanization, and family history of ASCVD were added to the equation.

### Definitions

In this study, educational level was divided into three types: primary school or less, middle school, high school or more. Family income was divided into three levels (China Yuan/year): low (≤ 5000), middle (5000–20,000) and high (> 20,000). As recommended by the Working Group on Obesity in China, obesity was defined as a body mass index (BMI) of 28.0 kg/m^2^ or higher [20]. In accordance with the JNC 7 Guidelines [21], hypertension was defined as a SBP ≥ 140 mmHg and/or a diastolic blood pressure (DBP) ≥ 90 mmHg and/or the use of antihypertensive medications. Diabetes mellitus was defined as a fasting blood glucose (FBG) ≥ 7.0 mmol/L, and/or being on treatment by the World Health Organization criteria [22]. The National Cholesterol Education Program-Third Adult Treatment Panel criteria was followed for defining dyslipidemia (one of the following elements: TC ≥ 6.21 mmol/L, HDL-C < 1.03 mmol/L, low density lipid cholesterol (LDL-C) ≥ 4.16 mmol/L and triglycerides (TG) ≥ 2.26 mmol/L) [23].

### Statistical analysis

Data were expressed as mean ± standard deviation, percentage, correlation coefficient and β. Differences between groups were compared using two-tailed Student’s t-test, variance analysis or *χ*^2^ test as appropriate. The mean levels of PHQ-9 score among different 10-year ASCVD risk categories by sex were calculated and presented. Univariate general lineal model was used to test the interaction of sex and 10-year ASCVD risk category for PHQ-9 score. Pearson correlation analysis was performed to investigate the correlations between 10-year ASCVD risk and PHQ-9 score by sex and different medical conditions. Univariate and multivariate linear regression analyses were both conducted to identify the crude and adjusted linear associations of sex and 10-year ASCVD risk with PHQ-9 score. Further, the potential interaction of sex and 10-year ASCVD risk on PHQ-9 score was tested. All statistical analyses were performed using SPSS 17.0 software (SPSS Inc., Chicago, IL, USA), and a *P* <  0.05 was considered as statistically significant.

## Results

### Characteristics of the study population

Of the 11,956 participants, 896 had incomplete data and were excluded from the analysis, leaving a total of 11,060 participants (5080 males and 5980 females) with a mean age of 53.9 years. Table 1 presented the sex-specific baseline characteristics of the study population. Differences between males and females were compared using two-tailed Student’s t-test or *χ*^2^ test as appropriate. As a result, the male subjects were significantly older than females (54.4 ± 10.8 vs. 53.4 ± 10.3; *P* <  0.001). They had significantly higher levels of SBP, DBP, FBG and education, lower levels of BMI, TC, LDL-C and income, and higher percentage of smoking and drinking (all *P*s <  0.05), whereas, there were no significant differences in TG and HDL-C between two groups. It’s worth nothing that males had significantly higher 10-year ASCVD risk than females (14.2 ± 10.7% vs. 9.3 ± 9.1%; *P* <  0.001) but lower level of PHQ-9 score (2.34 ± 3.13 vs. 3.63 ± 4.02; *P* <  0.001).

**Table 1:** Characteristics of the study sample

Variable	Male (*n* = 5080)	Female (*n* = 5980)	*P* value
Age, years	54.4 ± 10.8	53.4 ± 10.3	< 0.001
BMI, kg/m^2^	24.7 ± 3.5	24.9 ± 3.8	0.038
SBP, mmHg	143.5 ± 22.5	140.0 ± 24.0	< 0.001
DBP, mmHg	83.7 ± 11.7	80.6 ± 11.5	< 0.001
FBG, mmol/L	5.95 ± 1.63	5.87 ± 1.61	0.011
TC, mmol/L	5.17 ± 1.04	5.30 ± 1.12	< 0.001
TG, mmol/L	1.65 ± 1.62	1.62 ± 1.34	0.266
HDL-C, mmol/L	1.41 ± 0.42	1.41 ± 0.34	0.683
LDL-C, mmol/L	2.88 ± 0.79	2.97 ± 0.84	< 0.001
Current smoker	2906 (57.2)	980 (16.4)	< 0.001
Current drinker	2303 (45.3)	171 (2.9)	< 0.001
Education			< 0.001
≤ Primary school	2134 (42.0)	3405 (56.9)	
Middle school	2381 (46.9)	2110 (35.3)	
≥ High school	565 (11.1)	465 (7.8)	
Family income			0.016
Low	683 (13.4)	696 (11.6)	
Middle	2733 (53.8)	3292 (55.1)	
High	1664 (32.8)	1992 (33.3)	
PHQ-9 score	2.34 ± 3.13	3.63 ± 4.02	< 0.001
10-year ASCVD risk, %	14.2 ± 10.7	9.3 ± 9.1	< 0.001

*Abbreviations*: *BMI* body mass index, *ASCVD* atherosclerotic cardiovascular disease, *DBP* diastolic blood pressure, *FBG* fasting blood glucose, *HDL-C* high density lipid cholesterol, *LDL-C* low density lipid cholesterol, *PHQ-9* Patient Health Questionnaire-9, *SBP* systolic blood pressure, *TC* total cholesterol, *TG* triglycerides. Data are expressed as mean ± standard deviation or *n* (%)

### The sex-specific PHQ-9 score by 10-year ASCVD risk category

**Fig. 1:**
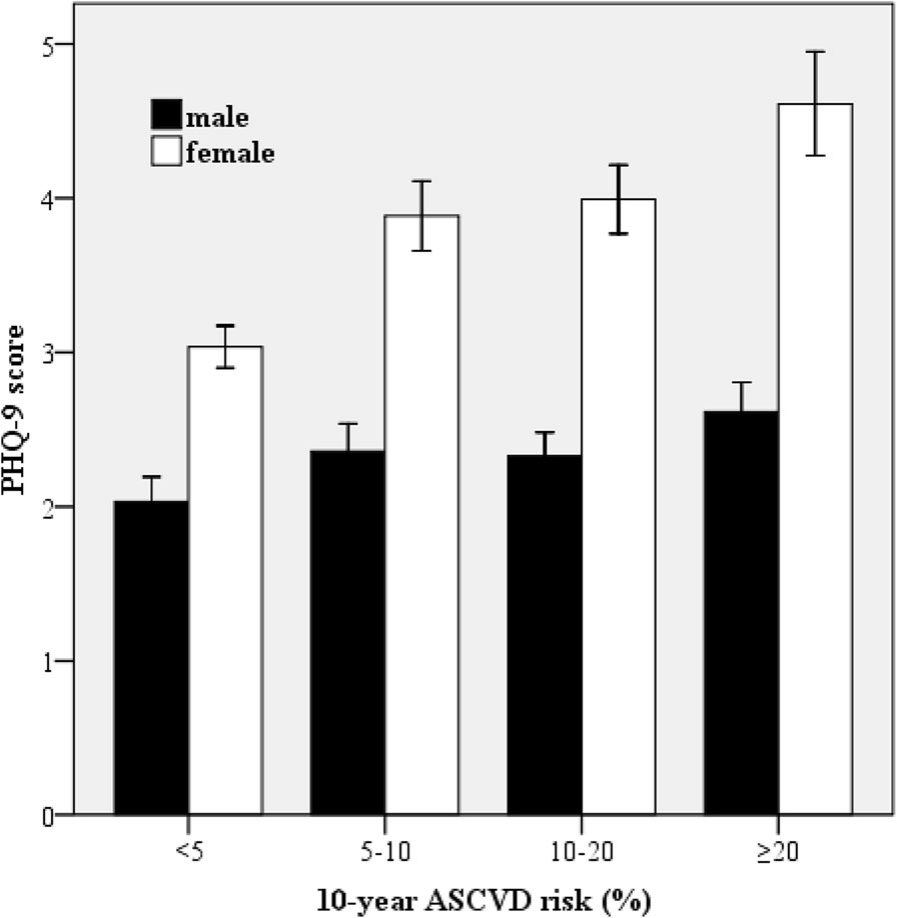
The sex-specific PHQ-9 score by 10-year ASCVD risk category. Error bars represent standard deviation. ASCVD = atherosclerotic cardiovascular disease; PHQ-9 = Patient Health Questionnaire-9

The mean levels of PHQ-9 score by sex and 10-year ASCVD risk category were presented in Fig. 1. Variance analysis was used to compare PHQ-9 score at different 10-year ASCVD risk categories. As a result, the mean PHQ-9 score increased significantly with advancing 10-year ASCVD risk category in both males (from the lowest of 2.03 to the highest of 2.61; *P* for trend < 0.001) and females (from the lowest of 3.04 to the highest of 4.61; *P* for trend < 0.001). Among each 10-year ASCVD risk category, the mean PHQ-9 score was significantly higher in females than males (all *P*s <  0.001). Further, the univariate general lineal model was used to test the interaction of sex and 10-year ASCVD risk category for PHQ-9 score, showing significant difference (*P* <  0.001).

### Pearson correlations between 10-year ASCVD risk and PHQ-9 score

The sex-specific pearson correlation analyses for associations between 10-year ASCVD risk and PHQ-9 score were conducted and presented in Table 2. In both sexes, 10-year ASCVD risk showed significant and positive correlations with PHQ-9 score (*P*s <  0.001). Further pearson correlation analyses presented various correlation coefficients according to different medical conditions (all *P*s <  0.05).

**Table 2:** Pearson correlations between 10-year ASCVD risk and PHQ-9 score

	Male		Female	
	Correlation coefficient	*P* value	Correlation coefficient	*P* value
All	0.079	< 0.001	0.131	< 0.001
Age, years				
< 60	0.051	0.003	0.087	< 0.001
≥ 60	0.062	0.015	0.064	0.009
Obesity				
Yes	0.126	< 0.001	0.141	< 0.001
No	0.074	< 0.001	0.136	< 0.001
Hypertension				
Yes	0.101	< 0.001	0.115	< 0.001
No	0.043	0.036	0.100	< 0.001
Diabetes				
Yes	0.095	0.033	0.082	0.036
No	0.064	< 0.001	0.120	< 0.001
Dyslipidemia				
Yes	0.116	< 0.001	0.133	< 0.001
No	0.045	0.011	0.121	< 0.001

*Abbreviations*: *ASCVD* atherosclerotic cardiovascular disease, *PHQ-9* Patient Health Questionnaire-9

### Linear relationship between 10-year ASCVD risk and PHQ-9 score

The univariate and multivariate linear regression analyses for associations of sex and 10-year ASCVD risk with PHQ-9 score were performed and presented in Table 3. Significant correlations of sex and 10-year ASCVD risk with PHQ-9 score were observed in univariate linear regression (all *P*s <  0.001). In the multivariate linear regression model, we included 10-year ASCVD risk, sex, and clinical covariates not in the 10-year ASCVD risk equation including BMI, DBP, TG, LDL-C, drinking, education and income. As a result, the independent association of 10-year ASCVD risk with PHQ-9 score remained in the total (β = 2.61; *P* <  0.001), male (β = 1.64; *P* = 0.001), and female subjects (β = 3.71; *P* <  0.001). The independent influence of sex on PHQ-9 score was also significant (*P* <  0.001). Finally, we tested the interaction of sex and 10-year ASCVD risk in both univariate and multivariate linear regression models, showing that sex had significant influence on the associations between 10-year ASCVD risk and PHQ-9 score with larger regression coefficients in females (*P*s <  0.001).

**Table 3:** Sex-specific linear regression analyses for associations between 10-year ASCVD risk and PHQ-9 score

	Model 1		Model 2	
	β	*P* value	β	*P* value
All				
Sex*	1.29	< 0.001	1.19	< 0.001
10-year ASCVD risk	2.17	< 0.001	2.61	< 0.001
Male				
10-year ASCVD risk	2.31	< 0.001	1.64	0.001
Female				
10-year ASCVD risk	5.81†	< 0.001	3.71†	< 0.001

*Abbreviations*: *ASCVD* atherosclerotic cardiovascular disease, *PHQ-9* Patient Health Questionnaire-9

Model 1: univariate linear regression model; Model 2: multivariate linear regression model including 10-year ASCVD risk, gender, body mass index, diastolic blood pressure, triglyceride, low density lipid cholesterol, drinking, education, and income

*: “0” for male and “1” for female in the analysis

†:*P* < 0.001 for gender difference

## Discussion

The results of this study indicated that the mean level of PHQ-9 score increased with advancing ASCVD risk category in both sexes, and the trend was more apparent in females than males. 10-year ASCVD risk positively correlated with PHQ-9 score with larger regression coefficients in females. Sex had significant effects not only on PHQ-9 score but also on the associations of 10-year ASCVD risk and PHQ-9 score. These findings firstly provide some data about associations between 10-year ASCVD risk and depressive symptoms in a general population.

Recent studies demonstrated that depression was positively associated with both CVD incidence among healthy individuals [24] and adverse cardiovascular events among patients with established CVD [25, 26]. Therefore, great efforts were conducted to make clear of the epidemiological characteristics of depression and to make population-based prevention strategies. Accordingly, depression was reported to be quite common among patients with CVD [27]. Similarly, the prevalence of depression was significantly higher in patients with heart failure [28], hypertension [29], diabetes [30], stroke [31] than healthy population. Thus, high prevalence of depression was presented among patients with CVD. Now, our data firstly indicated that depressive symptoms was more common in subjects with higher 10-year predicted risk of ASCVD, suggesting that screening and controlling of the potential depressive symptoms were needed among subjects with high risk of ASCVD.

The equation found by Gu et al. has been used widely as a tool suitable for China to assess the incidence risk of ASCVD, which was calculated based on age, treated or untreated SBP, TC, HDL-C, current smoking, diabetes mellitus, waist circumference, geographic region, urbanization and family history of ASCVD [5]. Previous studies have demonstrated that advancing age [7, 32], smoking [33], low HDL-C [34] and high SBP [28] were mostly correlated with depression although serum TC and depression might be inversely related [35]. Therefore, depression and ASCVD have some co-existing risk factors, which might partially explain the positive relationship between 10-year predicted risk and depressive symptoms in our current study. Further, health behaviors, inflammatory processes and heart rate variability might be the potential mechanisms that actually mediated the incidence of ASCVD and depression [36, 37].

However, some limitations are existing in our study. First, there was only PHQ-9 tool assessing depressive symptoms but no clinical diagnosis of depression by a psychiatrist. Second, the number of participants in some subgroups was relatively small so that an unintentional bias might be brought. Third, the current study was part of NCRCHS, and only rural Chinese subjects ≥35 years of age were included. In addition, covariates in the current study were relatively limited and some other possible covariates might give a bias.

## Conclusions

10-year ASCVD risk was positively associated with depressive symptoms in both males and females. And more apparent impacts of 10-year ASCVD risk on PHQ-9 score were observed in females. These findings provided some novel insights into the value of 10-year ASCVD risk in estimating depressive symptoms. Much attention should be paid to depressive disorders among subjects with high 10-year ASCVD risk.

## Abbreviations

ASCVD: atherosclerotic cardiovascular disease
BMI: body mass index
CHD: coronary heart disease
CVD: cardiovascular disease
DBP: diastolic blood pressure
FBG: Fasting blood glucose
HDL-C: high density lipid cholesterol
LDL-C: low density lipid cholesterol
PHQ-9: Patient Health Questionnaire-9
SBP: systolic blood pressure
TC: total cholesterol
TG: triglycerides
